# CONFIGURE: A pipeline for identifying context specific regulatory modules from gene expression data and its application to breast cancer

**DOI:** 10.1186/s12920-019-0515-6

**Published:** 2019-07-11

**Authors:** Sungjoon Park, Doyeong Hwang, Yoon Sun Yeo, Hyunggee Kim, Jaewoo Kang

**Affiliations:** 1 Department of Computer Science and Engineering, Korea University, Seoul, Republic of Korea; 2 Interdisciplinary Graduate Program in Bioinformatics, Korea University, Seoul, Republic of Korea; 3 Department of Biotechnology, School of Life Sciences and Biotechnology, Korea University, Seoul, Republic of Korea; 4 Institute of Animal Molecular Biotechnology, Korea University, Seoul, Republic of Korea

**Keywords:** Context specific regulatory module, Gene regulatory network inference, Single sample GSEA, Feature importance score, Breast cancer subtype

## Abstract

**Background:**

Gene expression data is widely used for identifying subtypes of diseases such as cancer. Differentially expressed gene analysis and gene set enrichment analysis are widely used for identifying biological mechanisms at the gene level and gene set level, respectively. However, the results of differentially expressed gene analysis are difficult to interpret and gene set enrichment analysis does not consider the interactions among genes in a gene set.

**Results:**

We present CONFIGURE, a pipeline that identifies context specific regulatory modules from gene expression data. First, CONFIGURE takes gene expression data and context label information as inputs and constructs regulatory modules. Then, CONFIGURE makes a regulatory module enrichment score (RMES) matrix of enrichment scores of the regulatory modules on samples using the single-sample GSEA method. CONFIGURE calculates the importance scores of the regulatory modules on each context to rank the regulatory modules.

We evaluated CONFIGURE on the Cancer Genome Atlas (TCGA) breast cancer RNA-seq dataset to determine whether it can produce biologically meaningful regulatory modules for breast cancer subtypes. We first evaluated whether RMESs are useful for differentiating breast cancer subtypes using a multi-class classifier and one-vs-rest binary SVM classifiers. The multi-class and one-vs-rest binary classifiers were trained using the RMESs as features and outperformed baseline classifiers. Furthermore, we conducted literature surveys on the basal-like type specific regulatory modules obtained by CONFIGURE and showed that highly ranked modules were associated with the phenotypes of basal-like type breast cancers.

**Conclusions:**

We showed that enrichment scores of regulatory modules are useful for differentiating breast cancer subtypes and validated the basal-like type specific regulatory modules by literature surveys. In doing so, we found regulatory module candidates that have not been reported in previous literature. This demonstrates that CONFIGURE can be used to predict novel regulatory markers which can be validated by downstream wet lab experiments. We validated CONFIGURE on the breast cancer RNA-seq dataset in this work but CONFIGURE can be applied to any gene expression dataset containing context information.

## Background

Many researches have identified biological phenotypes (i.e., contexts) such as cancer subtypes or cell types from gene expression data. Usually, clustering algorithms are applied to gene expression data for identifying biological contexts [1–3]. Though gene expression signatures accurately represent biological contexts from clustering results, it is difficult to identify the biological mechanisms underlying each biological context.

When context information is available, differentially expressed gene (DEG) analysis [4–6] is the most widely used for identifying marker genes that help to differentiate contexts. However, from DEGs, it is often difficult to identify the phenotypes or biological networks that are differentiated between the contexts. To overcome this problem, the gene set enrichment analysis (GSEA) method is widely used. The GSEA method can identify phenotypes or biological networks in which the DEGs are over-represented [7]. However, since the GSEA method is based on gene sets, the interaction information of biological networks is ignored. Several methods consider the interactions in biological networks when identifying context specific subnetworks [8–10]. However, these methods are unable to score subnetworks for single samples.

A gene regulatory network (GRN) describes transcriptional relationships between transcription factors (TFs) and their target genes. Among various data types, gene expression data is often used for inferring GRNs. The core component of the GRN inference method involves calculating regulatory interaction scores of genes; statistical and machine learning methods are applied for scoring interactions. However, most GRN inference algorithms are unsuitable to identify context specific GRNs [11–15].

Recently, the authors of [16] have developed a single cell GRN inference and clustering method called SCENIC. SCENIC was developed to infer GRNs of single cells and identify new cell types by clustering single cells based on the activity scores of the GRN modules. However, SCENIC does not prioritize GRN modules for each identified context of a cell type.

In this work, we present CONFIGURE which is a pipeline for identifying CONtext speciFIc reGUlatoRy modulEs. CONFIGURE first constructs regulatory modules from gene expression data using a gene regulatory network inference method and a transcription factor (TF) motif enrichment analysis method[13, 16]. A regulatory module consists of a TF and its target genes, and the regulatory interaction scores of them. Using the single sample gene set enrichment analysis (ssGSEA) method [17], CONFIGURE calculates the enrichment scores of all regulatory modules for all samples. An enrichment score indicates the degree of up- or down- regulation of a regulatory module for a given sample. To identify context specific regulatory modules, the importance scores of regulatory modules are computed on each context. To obtain the importance scores, CONFIGURE computes the feature importance scores of one-vs-rest binary random forest classifiers. The random forest classifiers are trained on each context using the enrichment scores of regulatory modules as features. Based on the feature importance scores computed by the random forest classifiers, CONFIGURE ranks regulatory modules on each context.

## Methods

### Input and output of CONFIGURE

**Fig. 1:**
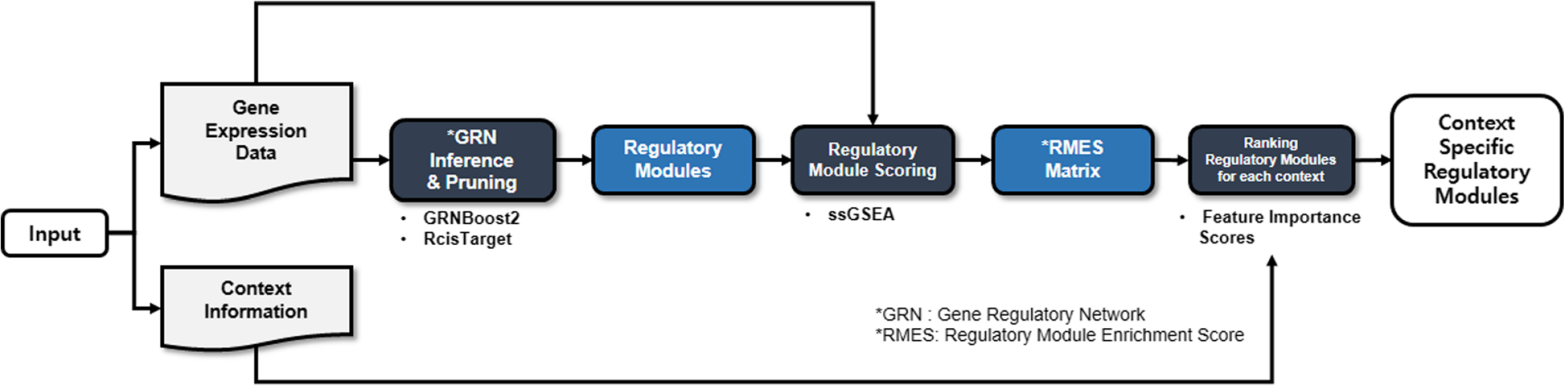
Overview of CONFIGURE

The overview of CONFIGURE is shown in Fig. 1. Gene expression data and context information are used as inputs of CONFIGURE. The gene expression data is a two dimensional matrix where samples and genes are listed in rows and columns, respectively. Entries of the matrix are gene expression values. The context information contains samples with their context labels. For each context, CONFIGURE outputs regulatory modules ranked based on their importance scores. We regard the regulatory modules with high importance scores in each context as the context specific regulatory modules.

To obtain the context specific regulatory modules, CONFIGURE performs the following three tasks: Constructing regulatory modules from gene expression data, constructing a regulatory module enrichment score (RMES) matrix, and computing importance scores of regulatory modules on each context. The following sections describe each task in detail.

### Construction of regulatory modules

**Fig. 2:**
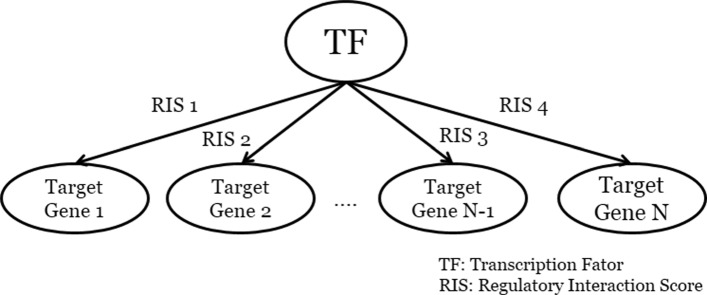
An illustration of a regulatory module

CONFIGURE constructs regulatory modules from gene expression data used as input. First, CONFIGURE constructs a gene regulatory network using GRNBoost2 which is a gene regulatory network inference method provided in SCENIC [16]. GRNBoost2 infers a gene regulatory network from gene expression data using a stochastic gradient boosting method [18]. The output of GRNBoost2 is a directed weighted network where a node indicates a TF or a target gene, and an edge indicates a regulatory interaction between a TF and a target gene. Then, the gene regulatory network is divided into regulatory modules using the modules_from_adjacencies function provided in the pySCENIC package [16]. We define a regulatory module as a tree with a depth of 1 where a root node is a TF and leaf nodes are target genes. The weight of an edge is the regulatory interaction score (RIS) which indicates the degree of regulation of a given target gene by a TF. Figure 2 illustrates a regulatory module. Regulatory modules are represented as either activated regulatory modules or repressed regulatory modules. Activated regulatory modules contain only interactions where the expression values of a TF are positively correlated with the expression values of target genes. If regulatory modules contain only negatively correlated interactions, they are repressed regulatory modules. Regulatory modules are further pruned using the RcisTarget method [16] which filters low confident target genes by motif enrichment analysis.

### Construction of a regulatory module enrichment score (RMES) matrix

After constructing regulatory modules, CONFIGURE constructs a regulatory module enrichment score (RMES) matrix. The RMES matrix contains the enrichment scores of samples and regulatory modules where samples are listed in the rows and regulatory modules are listed in the columns of the matrix. The enrichment score indicates the extent of up- or down-regulation of a given regulatory module in a given sample. The enrichment score is obtained using the single sample gene set enrichment analysis (ssGSEA) method [17].

The ssGSEA method computes the enrichment score of a given gene set for a single sample. The ssGSEA method and the original GSEA method are similar [7], but they use different gene score values. In the original GSEA method, gene score values are usually fold change of different contexts. However, in the ssGSEA method, gene score values of a sample are rank normalized where a gene with a high expression value is a high rank normalized value. Also, the ssGSEA method computes enrichment scores using the empirical cumulative distribution function (ECDF) whereas the GSEA method computes scores using the Kolmogorov-Smirnov statistic. We use normalized enrichment scores (NESs) as the entries of the RMES matrix.

### Computing importance scores of regulatory modules

To identify context specific regulatory modules, CONFIGURE uses the RMES matrix and context information of samples, and it computes the importance scores of the regulatory modules on each context. The importance scores are computed based on the feature importance scores of one-vs-rest binary random forest classifiers using RMESs as the features of the random forest classifiers [19]. Assuming we have a *C* number of contexts in context information {c _1_,c _2_,.. c _*C*_}, then the *C* number of binary random forest classifiers are trained. When training a binary random forest classifier on the context c _1_, samples with the context label “c _1_” are considered as positive samples, and samples without the context label “c _1_” are considered as negative samples. After training the random forest classifiers on each context, the feature importance scores of the classifiers are computed on each context. Feature importance scores of a random forest classifier are based on the average delta impurity scores of the base decision tree classifiers. The importance score of a regulatory module *m* using the feature importance scores (FIS) of a random forest classifier, which are based on the Gini impurity score, is calculated as follows [19–21]:

1 $$ {} Importance(m) = FIS({m}) =\frac{1}{T}\sum_{t=1}^{T}{\sum_{n: f(n)={m}}^{}{ \frac{{S}_{n}}{S} \Delta Gini(n) }}  $$

2 $$ {} \Delta Gini(n)= Gini(n)-\frac{{{S}}_{{n}_{left}}}{{S}_{n}}Gini\left({n}_{left}\right)-\frac{{S}_{{n}_{right}}}{{S}_{n}}Gini\left({ n }_{right} \right)  $$

3 $$ \\ Gini\left(n \right) \quad =\quad 1-\sum_{c=1 }^{C }{ { [p(c|n)]^{2}} } \\ \quad  $$

where *T* indicates the number of base decision trees in a random forest, *n* denotes a node in a base decision tree, *n*_*left*_ and *n*_*right*_ denote the left and right child nodes of *n*, respectively. *f*(*n*) indicates the feature used for splitting the node *n*. *S* is the total number of samples, *S*_*n*_ is the number of samples on node *n*, *C* is the total number of contexts, and *p*(*c*|*n*) is the probability of the samples having the context *c* on node *n*.

Since we are using RMESs as features, regulatory modules are given with feature importance scores. The importance score of a regulatory module indicates the degree to which the RMESs of the regulatory module have contributed in differentiating between positive and negative samples. We consider the regulatory modules with high feature importance scores for a given context as the context specific regulatory modules for that context. If the average RMESs of context specific regulatory modules are higher in positive samples, the modules are considered as up-regulated context specific modules; otherwise, they are considered as down-regulated context specific modules. We use the random forest classifier implemented in the scikit-learn Python machine learning package (RandomForestClassifier(n_estimators=500, criterion=’gini’)) [22].

## Results

### TCGA breast invasive carcinoma dataset

We tested whether CONFIGURE can produce biologically meaningful context specific regulatory modules using RNA-seq data from the Cancer Genome Atlas (TCGA) Breast Invasive Carcinoma (BRCA) dataset. Breast cancer can be divided into the following four subtypes: Luminal A, Luminal B, Her2, and basal-like [23, 24]. Table 1 lists the breast cancer types according to the expression status of breast cancer biomarkers [25]. Compared with other types, the basal-like type breast cancers have a poorer prognosis. Furthermore, it is difficult to find specific drug targets for the basal-like type breast cancers due to the absence of breast cancer biomarkers [26]. Identifying basal-like type regulatory modules can help to understand the regulatory mechanisms underlying basal-like type breast cancers and suggest new therapy options for such cancers.

**Table 1:** Classifying breast cancer subtypes according to the expression status of three breast cancer biomarkers

		ER	PR	HER2	Ki67
Luminal A		+	+	-	-
Luminal B	HER2 +	+	+	+	
	HER2 -	+	+	-	+
HER2		-	-	+	
Basal-like		-	-	-	

We downloaded the RNA-seq data of the TCGA BRCA dataset (provisional) from cBioPortal [27–29]. We used the “data_RNA_Seq_v2_expression_median.txt” file from the TCGA BRCA dataset which contains RNA-seq data for 1100 samples. The RNA-seq data was quantified using the RSEM method [30]. Since the subtype information of samples in the TCGA BRCA dataset was not provided, we used the PAM50 method [31] to produce subtype labels for all the samples in the TCGA dataset. We used the genefu R package for running the PAM50 method [32]. Among 1100 samples, 1072 samples were classified as Luminal A, Luminal B, Her2, or basal-like by using the genefu R package and theses samples were used for the analysis. Table 2 shows the number of samples in each breast cancer subtype used for the analysis.

**Table 2:** The number of samples of each breast cancer subtype

	Luminal A	Luminal B	Her2	Basal-like	Total
# of Samples	391	370	109	202	1072

### Construction of regulatory modules of breast cancer

Using the gene expression data from the TCGA BRCA dataset, we first constructed regulatory modules of breast cancers. The gene expression data was log2 normalized after adding 1 to all the gene expression values. We used a list of 800 transcription factors, which was obtained from the TRRUST database [33, 34]. Using the gene expression data and transcription factor list, a gene regulatory network of breast cancer is constructed using the GRNBoost2 method. After filtering edges with low weight values and further pruning by RCisTarget, regulatory modules of breast cancers are then constructed. A total of 110 regulatory modules with 34.682 target genes on average and a standard deviation of 23.476 were obtained.

### Quantitative evaluation

We first tested whether RMESs are useful for differentiating breast cancer subtypes. To do this, we performed 10-fold cross validation and evaluated the performance of the multi-class support vector machine (SVM) classifier which uses RMESs as features[35]. We used the scikit-learn Python implementation of the SVM classifier (LinearSVC(penalty=’l1’, multi_class=’ovr’,dual=False)) [22].

Table 3 shows the performance of the multi-class classifiers. Accuracy, F1-macro, and F1-weighted were used as the evaluation metrics. The accuracy score is defined by the number of correctly predicted samples divided by the total number of predicted samples. The F1-macro score is the average of the F1-scores of all contexts where F1-score is defined as follows.

$$F1-score = \frac{2\times Precision\times Recall}{Precision + Recall} \ where$$

$$Precision = \frac{\# \ of \, True \, Positives}{\# \, of \, True \, Positives + \# \, of \, False \, Positives }$$

$$Recall = \frac{\# \, of \, True \, Positives}{\# of \, True \, Positives \, + \# of \, True \, Negatives }$$

**Table 3:** Performance of multi-class classifiers

	Accuracy	F1-macro	F1-weighted
**SVM-RMES**	0.8983	0.8924	0.8986
SVM-Gene expression	0.8899	0.8917	0.8898
SVM-Gene expression (Hallmarks)	0.8834	0.8923	0.8831
COSSY	0.8657	0.8225	0.8723
Dominant Class Prediction	0.3451	0.1283	0.5132

The F1-weighted score represents the weighted average F1-scores where the support values of each context are weighted when averaging the F1-scores. We used the following four baseline classifiers: the multi-class SVM classifier using gene expression values as features (# of genes = 20531), the multi-class SVM classifier using gene expression values of cancer hallmark genes as features (# of genes = 167), the COSSY classifier, and the classifier that predicts the dominant class in the dataset (here, dominant class is Luminal A). The cancer hallmark genes were obtained from the COSMIC database [36]. The COSSY method identifies subnetworks that differentiate two contexts based on the entropy scores of the subnetworks[10]. Subnetworks with low entropy scores are ranked highly, indicating that the subnetworks accurately differentiate two contexts. The COSSY classifier predicts context labels based on weighted voting using highly ranked subnetworks. For the multi-class prediction, COSSY was trained on each breast cancer subtype in a one-vs-rest manner and the context with the highest positive weight was chosen. Table 3 shows the performance of the multi-class classifiers which were evaluated using 10-fold cross validation. The multi-class SVM classifier using RMESs as features achieved similar or slightly higher scores than the multi-class SVM classifier using gene expression values as features, and much higher performance than COSSY. COSSY is similar to CONFIGURE in that COSSY identifies context specific subnetworks. However, since classification is not the main purpose of COSSY, the classification scores may be low. The SVM classifiers trained using gene expression values as features achieve higher classification performance than COSSY as shown in Table 3 but they cannot identify context specific subnetworks. However, CONFIGURE can identify context specific subnetworks and achieve high classification performance.

We also evaluated the performance of one-vs-rest binary classifiers. One-vs-rest binary classifiers were trained on each subtype where samples of a given subtype were considered as positive samples and samples of other subtypes were considered as negative samples. Table 4 shows the accuracy score of each breast cancer subtype. The dominant class prediction classifier achieved an F1-score of 0 for all four subtypes because the negative class was the dominant class for all the subtypes, which resulted in 0 true positives. Our model which is the one-vs-rest binary SVM classifier trained using RMESs also obtained similar or slightly better performance than the SVM classifier trained using gene expression values. Also, our model obtained much better performance than COSSY. The classification results from the multi-class and one-rest-binary class experiments show that RMESs are useful features for differentiating contexts.

**Table 4:** Performance of one-vs-rest binary classifiers

	Luminal A	Luminal B	HER2	Basal-like	Average
	Accuracy				
**SVM-RMES**	0.9366	0.8722	0.9627	0.9907	0.9405
SVM-Gene expression	0.9104	0.8741	0.9664	0.9841	0.9338
SVM-Gene expression (Hallmarks)	0.9291	0.8657	0.958	0.9888	0.9354
COSSY	0.8871	0.7836	0.9067	0.9813	0.8897
Dominant Class Prediction	0.6353	0.6549	0.8983	0.8116	0.75
	F1-Score				
**SVM-RMES**	0.913	0.8105	0.7959	0.9747	0.8736
SVM-Gene expression	0.8772	0.8143	0.8378	0.958	0.8719
SVM-Gene expression (Hallmarks)	0.9033	0.8	0.7887	0.9698	0.8655
COSSY	0.8428	0.8542	0.3101	0.9506	0.7394
Dominant Class Prediction	0	0	0	0	0

### Validating basal-like type specific regulatory modules

Determining whether CONFIGURE can identify regulatory modules that can represent each context (here, breast cancer subtype) is crucial. Basal-like type breast cancer is a type of triple negative breast cancer where the expression status of Estrogen Receptor (ER), Progesterone Receptor (PR), and HER2 is negative. Basal-like type breast cancers usually have higher grade tumors and poorer prognosis than other subtype breast cancers. Due to their triple negative characteristic, there is a lack of targeted therapies for basal-like type breast cancers. Even with chemotherapies, it is difficult to dramatically improve the prognosis of patients with these cancers [23, 26, 28, 28]. Thus, it is essential to identify the transcriptional mechanisms underlying basal-like type breast cancers and eventually identify the molecular targets of basal-like type breast cancers.

After confirming the accuracy of the basal-like type binary classifier in Table 4, we extracted basal-like type specific regulatory modules using CONFIGURE. Table 5 shows the results of the basal-like type specific regulatory modules. The “+” sign in the regulatory module name indicates that the regulatory module is activated, and the “-” sign indicates that the module is repressed. We ranked the regulatory modules based on their importance scores. The top 10 regulatory modules and their scores are shown in Table 5. Target genes in a regulatory module are ranked based on their regulatory interaction scores (the top 5 target genes are shown in Table 5). The Status column indicates whether a basal-like type regulatory module is up-regulated or down-regulated. A regulatory module is up-regulated if its average RMES value is higher in the positive samples than in the negative samples.

**Table 5:** The results of basal-like type specific regulatory modules obtained by CONFIGURE

**Regulatory Module**	**Target Gene**	**FIS**	**Status**	**Evidence**
POU5F1(-)	TOX3	0.1328	down-regulated	[38, 39]
	RALGPS2			
	FUT8			
	HMGCR			
	FOXA1			
ZIC1(-)	XBP1	0.1072	down-regulated	
	OVOL1			
	SLC1A4			
	SMAD7			
	CNTN1			
RARA(+)	RARA	0.0827	down-regulated	
	STARD3			
	PLEKHH3			
	MAG			
	PCGF2			
E2F3(+)	E2F3	0.0667	up-regulated	
	ANP32E			
	GEN1			
	SYNCRIP			
	BEND3			
GATA6(-)	MAST4	0.058	down-regulated	[40]
	PDE6B			
	ROBO2			
	KIF5A			
	ABI2			
PHOX2B(+)	PHOX2B	0.0454	up-regulated	
	DDC			
	MSGN1			
	AKR1D1			
	FABP7			
GLI3(-)	PPIF	0.0447	up-regulated	[41]
	ELF5			
	ORAI1			
	POR			
	HMGA1			
ETV6(+)	PHB2	0.0346	up-regulated	
	NCAPD2			
	VANGL2			
	PLEKHA5			
	ETV6			
SRF(-)	PAIP2	0.0344	down-regulated	[37, 42]
	ERLEC1			
	NECAP1			
	SCRN3			
	ZFP62			
PLAGL1(-)	SLC25A17	0.0327	down-regulated	
	NPBWR2			
	PTK6			
	SYCE2			
	HN1L			

We validated the basal-like type specific regulatory modules obtained by CONFIGURE through literature surveys. We checked whether the TFs of regulatory modules were reported to have associations with the phenotypes of basal-like type breast cancers. The Evidence column in Table 5 indicates whether the TF of a regulatory module has been reported.

Interestingly, a recent study by [37] has showed that the expression of serum response factor (SRF) promotes the stemness of basal-like type breast cancers by activating Interleukin 6 (IL6) through binding to the Yes-associated protein (YAP). In our result, the SRF(-) regulatory module was ranked 9th (Table 5). The SRF(-) regulatory module is down-regulated which indicates that the regulatory module contains only target genes repressed by SRF and the target genes are down-regulated in the basal-like type. The target genes of the SRF(-) regulatory module that are over repressed by SRF may be novel candidates for promoting the stemness of basal-like type breast cancers.

## Conclusion

In this article, we presented CONFIGURE, a pipeline that identifies context specific regulatory modules from gene expression data. CONFIGURE infers and prunes a gene regulatory network to construct regulatory modules. CONFIGURE uses normalized enrichment scores obtained using the single sample GSEA (ssGSEA) method to score the regulatory modules for given samples and make a regulatory module enrichment score (RMES) matrix. The enrichment score indicates the extent to which a regulatory module is up- or down-regulated in a given sample. Then using the feature importance scores of a one-vs-rest binary random forest classifier, CONFIGURE identifies context specific regulatory modules.

We quantitatively evaluated CONFIGURE in the multi-class experiment and one-vs-rest binary class experiment using 10-fold cross validation. In the multi-class experiment, the multi-class SVM classifier trained using RMESs as features achieved an accuracy of 0.8983, an F1-macro score of 0.894, and an F1-weighted score of 0.8986. In the one-vs-rest binary experiment, the one-vs-rest binary SVM classifier trained using RMESs as features achieved accuracy scores of 0.9356, 0.8806, 0.9328, and 0.9907 on Luminal A, Luminal B, HER2, and basal-like, respectively. The multi-class and one-vs-rest binary SVM classifiers performed the best in the multi-class and one-vs-rest binary experiments, respectively. We validated the basal-like type specific regulatory modules through literature surveys. Compared with other breast cancer subtypes, basal-like type breast cancers have a poor prognosis and lack targeted therapies. Thus, it is important to identify the transcriptional mechanisms underlying basal-like type breast cancers. The literature survey result showed that basal-like type specific regulatory modules are associated with the phenotypes of basal-like type breast cancers.

Although CONFIGURE has many advantages, there is still room for improvement. CONFIGURE can be applied to other types of cancer or any gene expression dataset as long as it contains contextual information (e.g., single cell RNA-seq data that contains cell type information). However, we validated CONFIGURE only on the breast cancer dataset. In future work, CONFIGURE can be validated on other datasets, and more importantly, regulatory modules identified by CONFIGURE can be verified through wet-lab experiments.

We believe that CONFIGURE will prove to be a useful pipeline for generating hypotheses about novel transcriptional mechanisms that accurately characterize phenotypes.
